# Fate of artificial sweeteners through wastewater treatment plants and water treatment processes

**DOI:** 10.1371/journal.pone.0189867

**Published:** 2018-01-02

**Authors:** Shaoli Li, Yuhang Ren, Yingying Fu, Xingsheng Gao, Cong Jiang, Gang Wu, Hongqiang Ren, Jinju Geng

**Affiliations:** State Key Laboratory of Pollution Control and Resource Reuse, School of the Environment, Nanjing University, Jiangsu, PR of China; Purdue University, UNITED STATES

## Abstract

Five full-scale wastewater treatment plants (WWTPs) in China using typical biodegradation processes (SBR, oxidation ditch, A^2^/O) were selected to assess the removal of four popular artificial sweeteners (ASs). All four ASs (acesulfame (ACE), sucralose (SUC), cyclamate (CYC) and saccharin (SAC)) were detected, ranging from 0.43 to 27.34μg/L in the influent. Higher concentrations of ASs were measured in winter. ACE could be partly removed by 7.11–50.76% through biodegradation and especially through the denitrifying process. The A^2^/O process was the most efficient at biodegrading ASs. Adsorption (by granular activated carbon (GAC) and magnetic resin) and ultraviolet radiation-based advanced oxidation processes (UV/AOPs) were evaluated to remove ASs in laboratory-scale tests. The amounts of resin adsorbed were 3.33–18.51 times more than those of GAC except for SUC. The adsorption ability of resin decreased in the order of SAC > ACE > CYC > SUC in accordance with the pKa. Degradation of ASs followed pseudo-first-order kinetics in UV/H_2_O_2_ and UV/PDS. When applied to the secondary effluent, ASs could be degraded from 30.87 to 99.93% using UV/PDS in 30 minutes and UV/PDS was more efficient and economic.

## Introduction

Artificial sweeteners (ASs) are popular sugar substitutes used in food, beverages, pharmaceuticals and personal care products [1]. Among them, acesulfame (ACE), sucralose (SUC), cyclamate (CYC) and saccharin (SAC) are allowed by law and consumed in large quantities in China. ASs have been listed as emerging contaminants, and ACE is among the anthropogenic trace contaminants with the highest concentrations in ground water and surface water as well as drinking water [2].

Wastewater treatment plants (WWTPs) are a source of ASs entering the aqueous environment. Surveys of this occurrence have become plentiful since the first research reporting the existence of SUC in rivers and WWTP effluents [3]. Concentrations of ASs from nano- to microgram levels in wastewater influents and effluents have been reported in European countries [2, 4–6] and in the USA [7], with variations between countries. While reports in Asian countries are limited, CYC and SAC have been reported as the dominant ASs with concentrations up to 41.877μg/L in Singapore [8] and ranging from 1.9 to 21μg/L in WWTP influent in Tianjin, China [1]. Lange et al. [2] reported that ACE and SUC were not biodegraded through WWTPs, while Falas et al. [9] recently studied the biodegradation of ACE through the sequencing batch reactor (SBR) process. So, it makes sense to trace the fate of ASs through different biodegradation processes and subsequent advanced treatment processes in WWTPs.

Although ASs have been approved as safe for human use, the eco-toxicity of ASs remains unclear. SUC has been extensively tested and may cause significant feeding and behavioral effects in crustaceans [10]. Reports regarding other ASs are scarce. ASs can cause developmental toxicity to *Oryzias latipes* [11]. Under prolonged UV photolysis, ACE and SUC transfer into intermediates that are more toxic [12]. Our previous report also found that the UV irradiation products of ACE can cause serious oxidative damage to *Carassius auratus* [13]. To minimize the side effects of ASs in the environment, advanced treatment processes in WWTPs are urgently needed.

Even though the removal of ASs is of extreme importance, not many studies can be found on ways to remove them when compared with other emerging contaminants. Granular active carbon (GAC) and resin are frequently used to adsorb emerging pollutants [14]. Mailler et al. [5] reported a moderate decrease of SAC and ACE by GAC adsorption. To the best of our knowledge, no research on using resin to remove ASs has been done. Besides adsorption, disinfection (such as chlorination, UV irradiation and ozonation) and advanced oxidation processes (AOPs) (such as Fenton, photo-Fenton, TiO_2_ photocatalysis, and UV/H_2_O_2_) are among the most applied and studied advanced treatment technologies aimed at improving the quality of the secondary effluent before disposal or reuse [15]. Regular UV irradiation has proved efficient only for removing ACE [16]. UV-AOP is more effective at removing ASs. SUC can be efficiently removed by UV/H_2_O_2_ and UV/TiO_2_ [17,18]. UV/PDS can entirely mineralize SUC [19]. ACE can be degraded by UV/TiO_2_ with the risk of increasing toxicity [12]. Few reports on the simultaneous degradation of the four common ASs are available. Furthermore, research comparing adsorption and AOP for removing ASs is limited, even at the laboratory scale.

In this study, the distributions of four ASs (ACE, SUC, SYC and CYC) and their treatment by five full-scale municipal wastewater treatment processes in China were investigated. Also, the fate of all four ASs through adsorption and UV/AOP treatment was assessed. The purposes of this study are as follows: (1) to evaluate the variation of four ASs throughout the wastewater treatment process with different treatment technologies in five full-scale WWTPs; (2) to compare the adsorption efficiency of ASs by GAC and resin in the laboratory; and (3) to compare UV/PS with UV/H_2_O_2_ treatment about degradation of ASs in the laboratory. The results provide a scientific basis for the controlling of ASs in wastewater biological and advanced treatment systems.

## Materials and methods

### Chemicals and reagents

Standard chemicals and regents for ACE, CYC, SAC and SUC were purchased from Sigma-Aldrich (St. Louis, MO, USA). Internal standard acesulfame-d4 was obtained from J&K Scientific, Ltd. The ion pair reagent tris (hydroxymethyl) aminomethane (TRIS) was obtained from Sigma-Aldrich. HPLC-grade acetonitrile was obtained from Merck (Darmstadt, Germany). Sodium peroxydisulfate (PDS) (Na_2_S_2_O_8_, 98%), H_2_O_2_ (analytical grade, 30% w/w) and GAC were purchased from Nanjing Chemical Reagent Company. The authors also used a magnetic anion exchange resin (NDMP), which was supplied by Shuang et al [14]. Milli-Q water, with a resistivity of at least 18.2 MΩ cm, was produced using a Millipore purification system (Billerica, CA, USA). All other solvents and reagents were of analytical grade.

### Sample campaign and sample extraction

**Fig 1:**
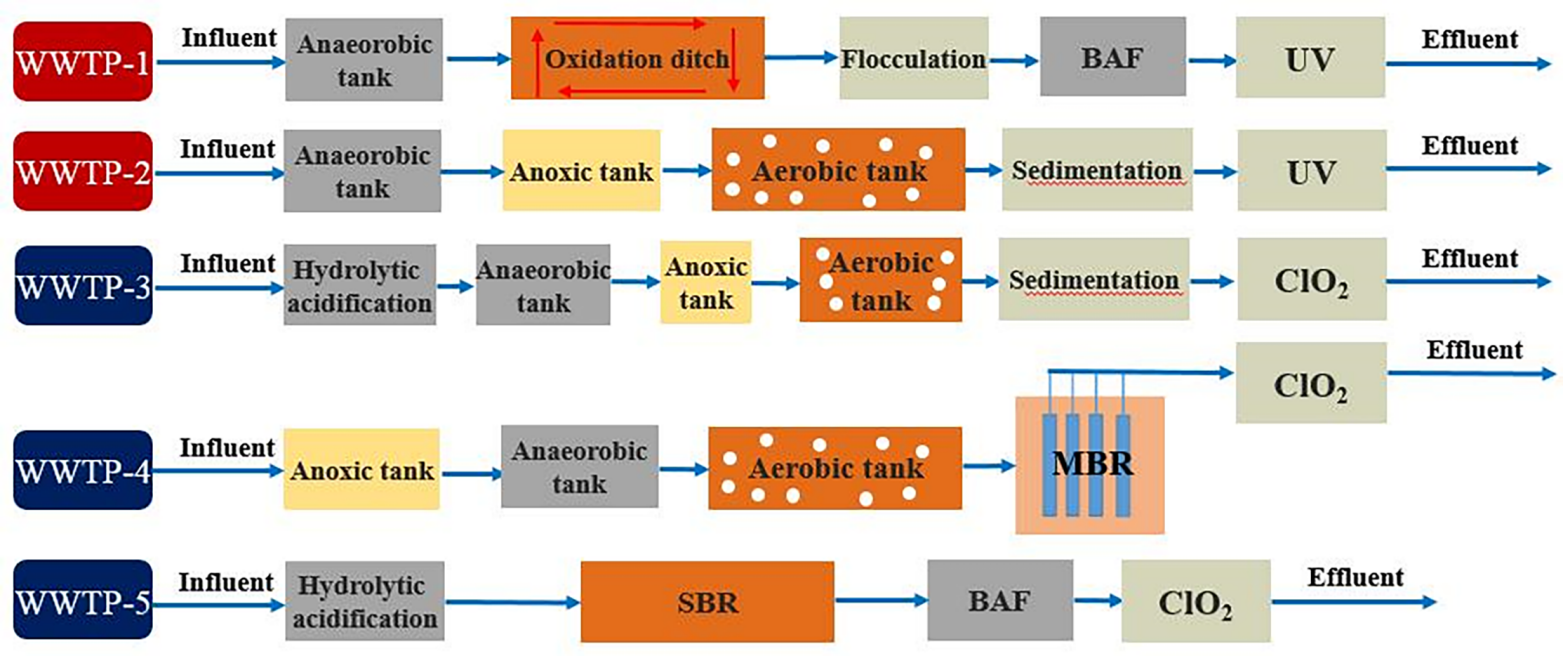
Schematic diagram of the investigated WWTPs.

To investigate the removal of target ASs in five full-scale WWTPs, wastewater samples were taken from routine WWTP sampling points at two WWTPs located in Nanjing and three in Wuxi, China. We obtained permission for sampling these locations, and the sampling was carried out in collaboration with each WWTP staff. The sampling campaign was conducted four times over a period of one year, on October 15^th^, 2015, and January 15^th^, April 15^th^, and July 15^th^, 2016. The data from October and January represent winter, and the data from April and July represent summer. These WWTPs treat both municipal wastewater and industrial wastewater. Industrial wastewater is pretreated to meet the discharge standard before it enters the municipal WWTPs. Detailed process parameters of the investigated WWTPs are shown in S1 Table. And Fig 1 describes schematic diagram of the investigated WWTPs process. All samples were grab samples. Raw wastewater samples were preserved in darkness at 4 ^o^C until analysis.

Wastewater samples were filtered using 0.45-μm cellulose nitrate membrane filters. The physico-chemical properties (COD, NH_4_^+^-N and TN and pH) of wastewater from sample sites are shown in S2 Table. The target ASs were concentrated by a solid phase extraction (SPE) method adopted from the literature [20]. CNW Poly-Sery PWAX cartridges (CNW Technologies GmbH, Düsseldorf, Germany) were used for SPE.

### Laboratory-scale ASs removal experiments

NDMP resin and GAC were used for adsorption studies at 293 K. For this purpose, 50 mL of ASs solutions with different concentrations (100, 500, 1000μg/L) were shaken at 130 rpm at 293 K with 3 mg of resin or GAC. The adsorption capacity was calculated using:

$$q_{t}=V\left(C_{0}\text{-}C_{t}\right)/W$$ (1)

where *q*_*t*_ (mg/g) is the adsorption amount, *V* (L) is the volume of solution, and *W* (g) is the weight of resin or GAC. C_0_ and C_t_ (mg/L) represent the initial concentration and concentration at time t (min), respectively.

UV irradiation and UV-based AOPs of ASs were conducted in a photoreaction operation reactor (XPA-7, Nanjing Xujiang Motor Factory, China). A low-pressure mercury light (22 W, 254 nm) was placed in the center of the reactor with a quartz cover. The light was supplied by an electronic ballast. The UV irradiation intensity was 520 uW/cm^2^, and the intensity was measured by a UV radiation meter (purchased from the Photoelectric Instrument Factory of Beijing Normal University, China). The irradiated sample was placed in a 50-mL quartz tube filled with ASs solution and stirred by an electromagnetic stirrer. A 0.1 mg/L mixed ASs solution and ACE solutions with different initial concentrations were UV irradiated. While the AOP was conducted, specific oxidants (PDS or H_2_O_2_) were first added to the irradiated sample and then UV irradiated. Samples were taken at specific time intervals within 30-minute UV irradiation periods.

Both UV/AOPs were further evaluated with secondary effluent sampled in WWTP-1. The reaction time and the amount of oxidant (PDS or H_2_O_2_) was the same as for pure water. The wastewater samples were also processed by SPE before analysis by LC-MS.

### Sample analysis

COD, NH_4_^+^-N, and TN were determined according to standard methods for the examination of water and wastewater (APHA, 2005). DO concentration, pH and temperature values were measured using oxygen (SG6, METTLER TOLEDO Inc., USA) and pH meters (FE20, METTLER TOLEDO Inc., USA).

The target ASs were analyzed using a Waters Acquity UPLC Tandem Waters Xevo TQ-S MS/MS system (Waters, USA) with an electrospray ionization (ESI) interface operated in multiple-reaction monitoring (MRM) mode. Detailed information on the parameters for MRM acquisition can be found in S3 Table. The negative ionization mode was set at -2 kV capillary voltage. The source and desolvation temperatures were set at 150 and 300°C, respectively. Nitrogen was used with a cone gas flow of 150 L/h for nebulization and a gas flow of 600 L/h for desolvation.

Chromatographic separation was performed with an Acquity UPLC BEH C18 column (2.1 × 50 mm, 1.7 um) at 30°C in gradient elution mode. An injection volume of 20μL was used for all samples. The mobile phase was composed of water (A) and acetonitrile (B), both containing 5 mM ammonium acetate and 1 mM TRIS. Gradient elution was carried out at a flow rate of 0.1 mL/min, and the mobile phase gradient was ramped linearly from 5% to 75% B in 3 min, returned to 5% within 1 min, and the system was allowed to equilibrate for 2 min before the next injection.

### Quality assurance and quality control

An eight-point calibration curve was established for the range 0.05~1000 μg/L. The instrumental detection limit (IDL) was determined by the minimum detectable amount of ASs for a signal-to-noise ratio of 3. The method detection limit (MDL) was determined by the lowest detectable concentration spike in wastewater giving a signal-to-noise ratio of 10, and the calculation of MDL took into consideration the concentration times. Detailed information on the Qa/Qc data is shown in S4 Table.

Recovery tests were conducted by spiking the ASs at certain concentrations in wastewater samples before extraction and comparing with non-spiked samples after the same whole extraction process. In addition, method precision and method accuracy were determined by six repeated injections of the same water sample during the same day (repeatability) and six injections on five different days (reproducibility). As can be seen in S4 Table, satisfactory recovery ranged from 89.91 to 93.73% in wastewater, and the relative standard deviations (RSD, %) were all below 6%. The repeatability and reproducibility of the method for ASs were 2.7~4.5% and 7.2~11.3%, respectively.

### Statistical analysis

Statistical analyses were performed using the SPSS statistical package. All data were determined at least three times and expressed as mean values ± standard deviation (SD). The significant differences between two experimental groups were compared by a one-way analysis of variance (ANOVA), and significance was identified by a post hoc LSD test at *p* < 0.05. Principle component analysis (PCA) was performed using Canoco software (Version 4.5). Curve-fitting equations were fitted using Origin (Version 9.0).

## Results and discussion

### Variation of ASs in WWTPs through different processes

#### Occurrences of ASs in influent and effluent

**Fig 2:**
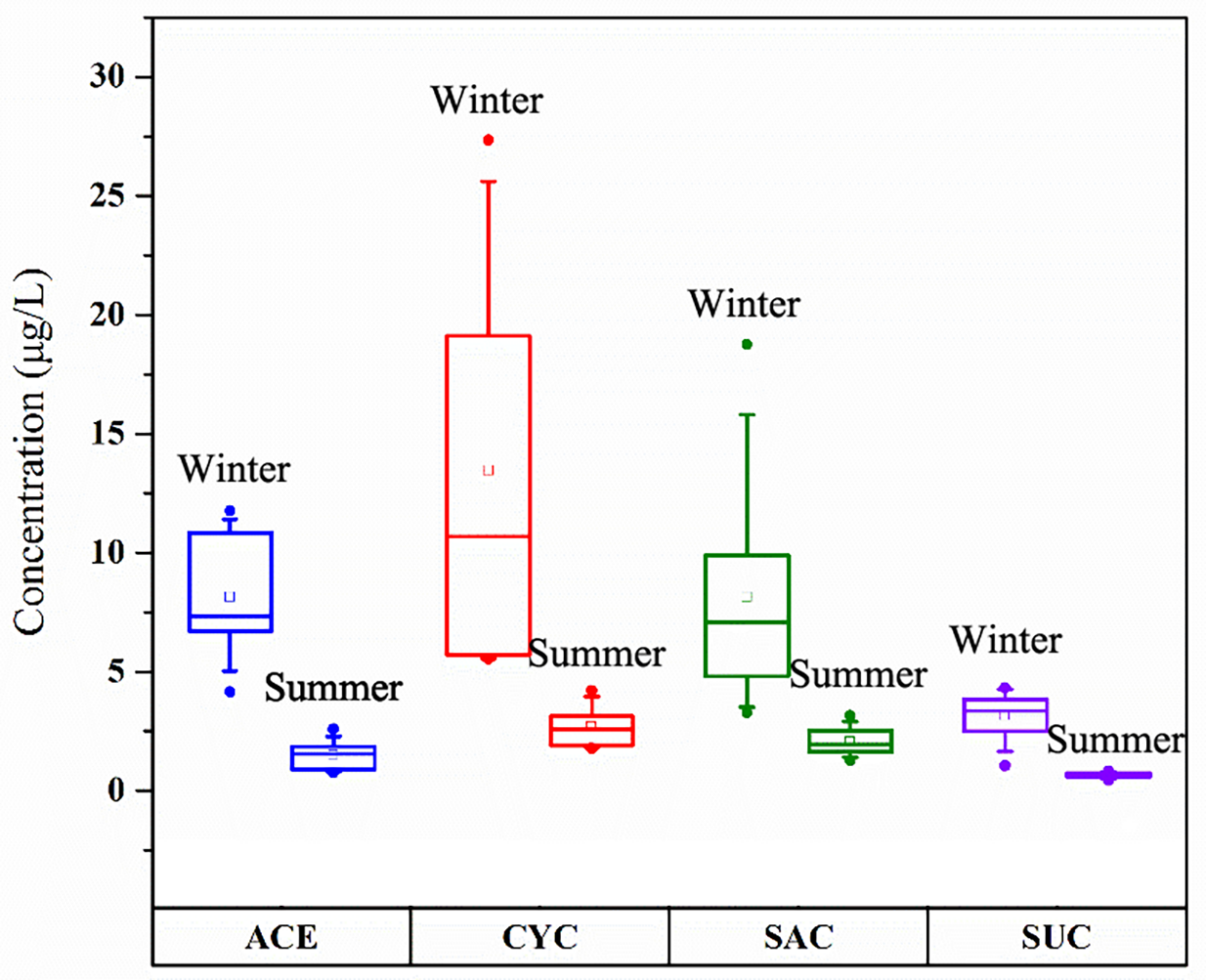
**Nonparametric probability distribution of the four ASs in WWTP influent** (The top and bottom of each box represent the 75th and 25th percentiles, respectively; the top and bottom of each whisker represent the 90th and 10th percentiles, respectively; the line inside the box represents the 50th percentile; the small square represents the mean value, and the small circle represents the max and min values).

The concentrations of four targeted ASs were detected in influent at levels of 0.43~27.34μg/L and decreased in the order CYC > ACE ≈ SAC > SUC (Fig 2), which was similar to findings in China and Singapore [1,8]. The concentrations of ASs in influent in winter exceeded those in summer by factors of 4.3~6.3, which is different from a former study in which CYC and SAC concentrations were higher in winter and ACE and SUC concentrations were higher in summer in an open coast system in HK [12]. This phenomenon may be explained by consumption habits and lower removal efficiency by biodegradation in WWTPs in winter.

The concentrations of CYC and SAC in effluent changed from 0.24 to 0.02μg/L; thus, they were almost entirely removed by biodegradation. The percentage of ACE removed was between -3.02% and 30.5%, while the concentration of SUC increased along the wastewater treatment process with removal percentages of -13.59%~-79.29%. This negative removal of SUC was also found by Brorström-Ludén et al [3]. One explanation for the negative removal could be SUC’s ability to conjugate with the enzymatic cleavage of glucuronide and, thus, release SUC in subsequent processes [21]. As for ACE, a controversial broad range of removal was reported in earlier publications. Most publications report that ACE can only be removed at a rate of less than 20% in WWTPs [2]. However, with a report of ACE removal of up to 97% in a nitrifying/denitrifying SBR [9, 22], its biodegradation ability remains open to further research.

#### Fate of ASs through biological treatment and physico-chemical processing

**Fig 3:**
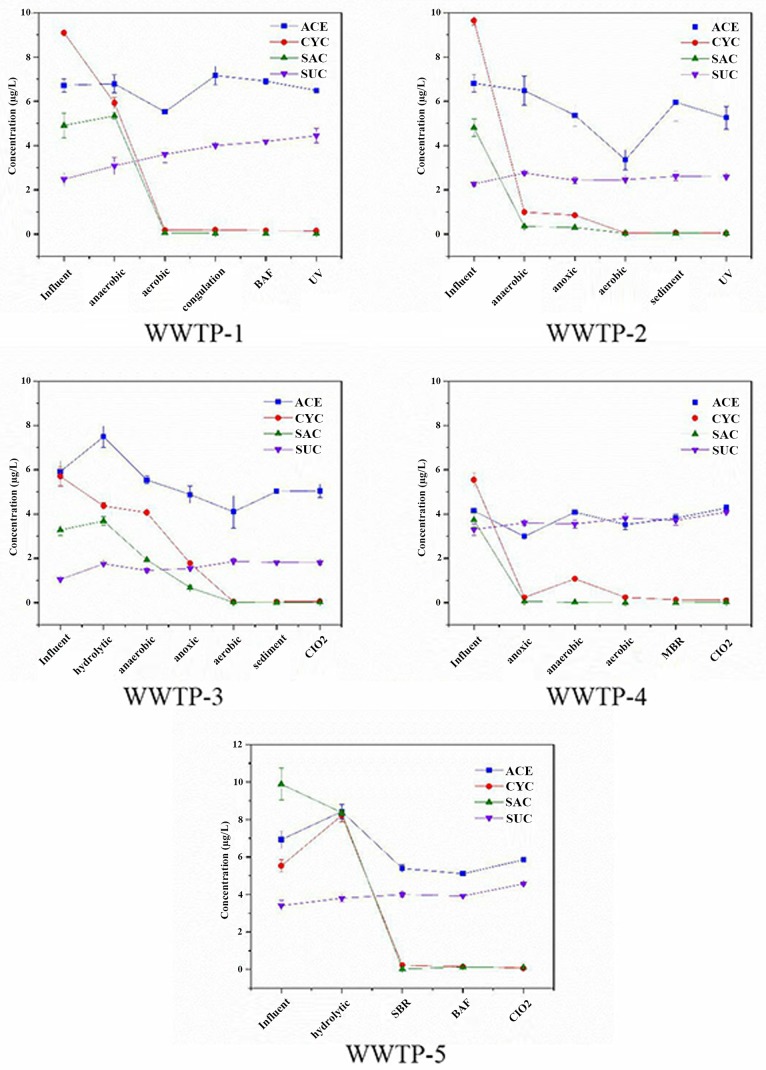
**Concentration of ASs through the treatment process in October in five WWTPs** (Detailed data for other months are shown in S5 Table).

The concentration of ASs through the treatment process in October in five WWTPs is shown in Fig 3 (detailed data for other months are shown in S5 Table). For biodegradable CYC and SAC, different biodegradation processes had no significant influence on their mean removal, which was over 96.3% throughout the year in all cases. The extremely persistent sweetener, ACE, was found to be partly eliminated in the biological treatment units, with the A^2^/O process giving a better performance (17.78–32.88%) than other biological processes (13.23–19.57%) (Table 1). In this study, the highest removal occurred in the anoxic process, suggesting that denitrification under anoxic conditions represents the major part of the degradation of ACE. It was deduced that ACE removal was associated with the nitrification process [23]. Castronovo et al. [22] confirmed that both oxic and denitrification contributed to the removal of ACE. In addition, the removal of ACE in WWTP-4 was the lowest of the three WWTPs using the A^2^/O process. The anaerobic process in the reversed A^2^/O in WWTP-4 induced the concentration of ACE (negative removal in Table 1). To control emerging contaminants like ACE, the anaerobic-anoxic-aerobic process seemed to be the better choice.

**Table 1:** Mean removal (%) of ACE (*n* = 4) for different treatment technologies.

	*Biological treatment*				*Disinfection*
WWTP-1	Anaerobic	Aerobic	BAF	Total	UV
	1.1±4.17	14.84±5.66	2.53±1.89	13.23±1.41	3.95±2.27
WWTP-2	Anaerobic	Anoxic	Aerobic	Total	UV
	3.65±2.06	17.08±0.84	12.15±6.84	32.88±12.81	5.62±5.57
WWTP-3	Anaerobic	Anoxic	Aerobic	Total	ClO_2_
	3.95±1.96	14.75±4.98	9.04±3.42	27.74±7.73	-0.55±2.24
WWTP-4	Anoxic	Anaerobic	Aerobic	Total	ClO_2_
	18.54±7.59	-17.03±7.14	16.27±6.51	17.78±9.81	-5.68±4.39
WWTP-5	SBR		BAF	Total	ClO_2_
	15.54±7.43		4.03±1.77	19.57±8.79	-17.17±6.49

In this study, MBR in WWTP-4 was not efficient at removing the targeted ASs (Fig 3), while MBR proved to be a better way to remove other emerging contaminants. Tran et al. [8] also observed that the MBR permeate did not contribute to the removal of ASs in WWTPs in Singapore. In recent years, BAF has been used after the biological process dedicated to removing micropollutants, but it does little work toward degrading ACE and SUC (Table 1).

Different physico-chemical processes performed various removal tasks with respect to these four ASs. For example, the concentration of some ASs bounced back in the subsequent sediment or disinfection unit (Fig 3). ASs such as SAC and ACE are polyfunctional ligands and offer different coordination sites to heavy metals and other organics [24]. So, their intermediates may complex with other materials and dissociate in a later process, which results in low removal in the effluent. UV disinfection was only efficient at removing ACE, with a removal rate between 1.23 and 11.89%. This minimal removal is due to limited UV irradiation in wastewater disinfection [25]. ACE was found to be degraded by 30% in the UV process in a German full-scale waterworks [16]. However, ClO_2_ disinfection does not degrade ASs in WWTPs [26]. The increasing concentrations of all four ASs in ClO_2_ disinfection may be due to the existence of precursors [16].

**Fig 4:**
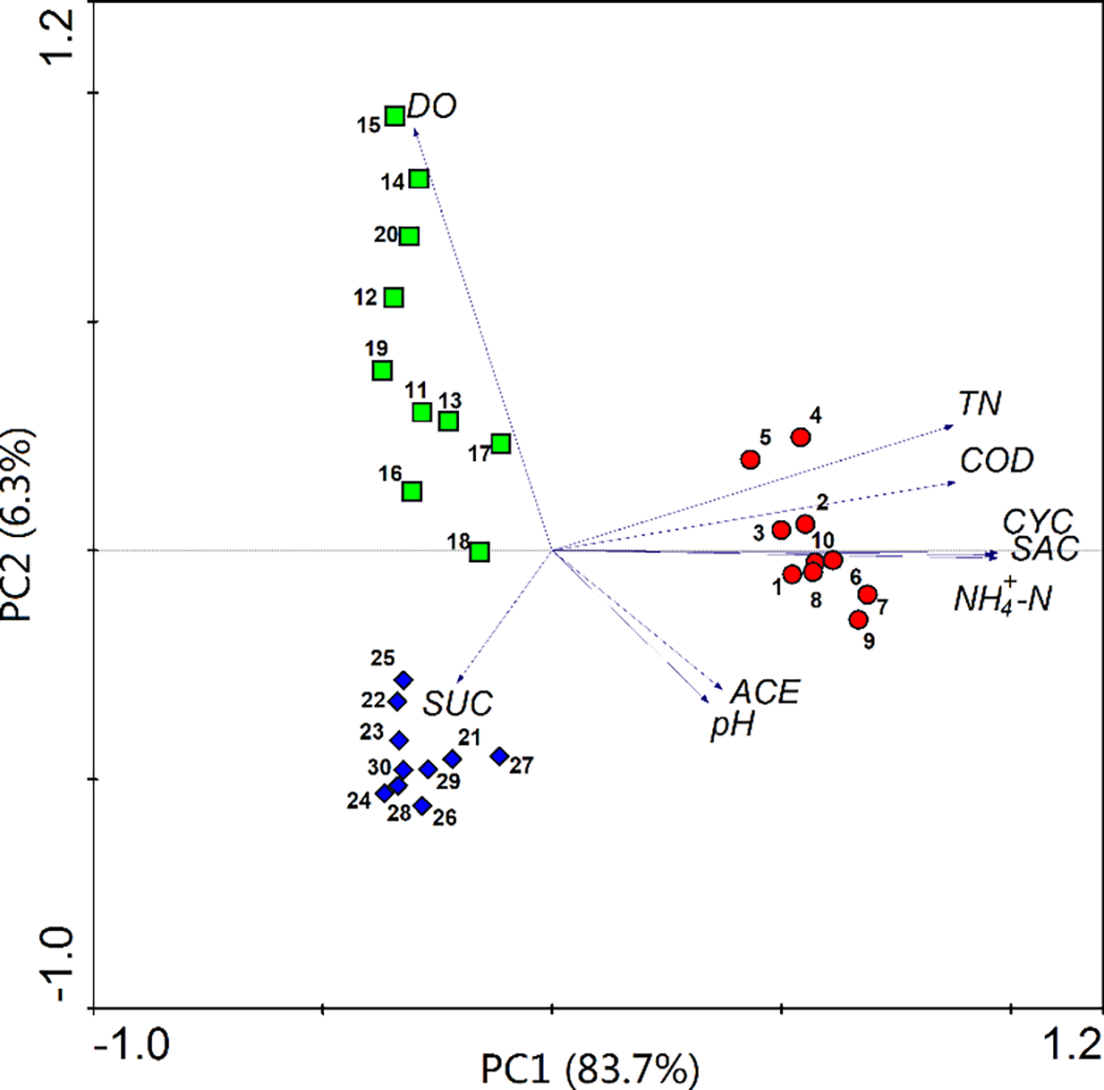
PCA analysis of ASs concentration and other physico-chemical indexes (data from winter) in influent (red circle, 1–10), secondary effluent (green square, 11–20) and effluent (blue diamond, 21–30). Each object is a data vector of the 9 following variables: pH, DO, COD, NH_4_^+^-N, TN, ACE, CYC, SAC and SUC.

To investigate the difference between sample sites and the relationship between AS concentrations and other physico-chemical indexes, PCA was performed and the results are shown in Fig 4. The distance between symbols reflects dissimilarity, and the intersection angle between arrows reflects the relationship between indexes [27]. CYC and SAC shared a similar degradation trend in WWTPs and had a positive correlation with wastewater parameters (TN, NH_4_^+^-N and COD), which indicated that they could be removed well. The symbols can be projected perpendicularly onto the arrows, showing abundance. Fig 4 shows that ACE concentrations in different WWTPs were similar in influent and effluent, while the concentrations were significantly reduced in biological processes (green squares), showing that different biological process varied in treatment efficiency. To summarize, the existing treatment processes did not prevent the emission of ASs, especially ACE and SUC, into an aquatic environment.

### Removal of ASs by GAC and resin

**Fig 5:**
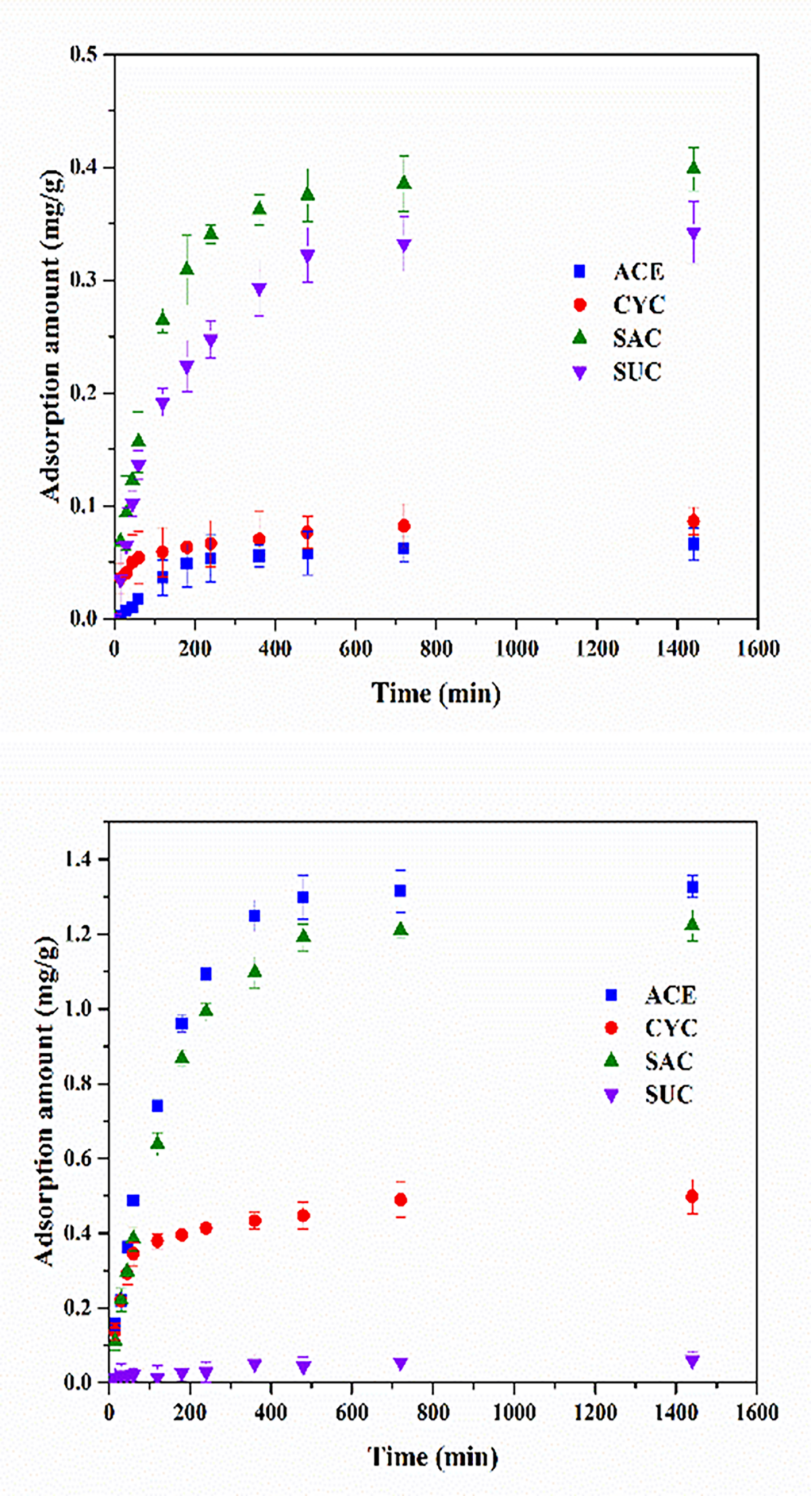
Adsorption of ASs by GAC and resin (C_0_ = 100 μg/L).

Considering the potential environmental risk caused by ASs, advanced treatment processes such as adsorption, UV and advanced oxidation should be applied to remove ASs. Adsorption by GAC or resin is competing methods for separating micropollutants in the wastewater advanced treatment process [14]. Fig 5A presents the adsorption ability of ASs on GAC. The amount of SAC adsorbed was the highest among the four ASs (0.4 mg/g), and the others are in the order of SUC > CYC > ACE. This is in accordance with Lange et al. [2], who determined that SAC had the highest octanol-water partition coefficient (i.e., SUC > CYC > ACE). Mailler et al. [5] found that up to 54% and 26% of SAC and SUC, respectively, could be removed when using a full-scale GAC filter. However, Scheurer et al. [28] reported negligible effects for ACE and CYC in their laboratory-scale experiment. For Soh et al. [29], the adsorption ability of SUC to GAC proved higher than ACE, but both of them are less likely to be adsorbed than chlordane, naphthalene and other contaminants.

In this research, resin was adopted to remove ASs and its ability was compared with that of GAC. NDMP resin was chosen due to its positive charge group and an outstanding ability to adsorb contaminants with a negative charge (such as ASs) through anion exchange. For the same amounts of GAC and resin adsorbent, the adsorption amounts of ASs using resin were 3.33–18.51 times higher than those of GAC, except for SUC (Fig 5B). The saturated adsorption amount decreased in the order of SAC > ACE > CYC > SUC, and it is generally in accordance with pK_a_. The poor adsorption of SUC to resin was due to its low ability to ionize. Regarding saturation, the removal of ASs was 79.54% (ACE), 31.9% (CYC) and 8.1% (SAC). Compared with GAC, better removal using NDMP resin was reported when treating DOM and acidic organics in dye wastewater [30]. The adsorption amounts of GAC and resin both increased with the initial ASs concentration (500 and 1000μg/L, as shown in S1 Fig).But no linear correlation was found between C_0_ and adsorption amounts.

In order to validate the results of ASs removal by GAC and resin under more realistic conditions, adsorption amount of ASs by GAC and resin was predicted in five full-scale WWTPs by curve-fitting equations (S6 Table).The curve-fitting equations were calculated according to the adsorption ability of ASs on GAC and resin at 100 μg/L ASs (Fig 5). The results were shown in S7 Table, and the relative coefficient was more than 0.879, indicating equations were well fitted. From S6 Table, we observed that adsorption amounts of ASs using resin were higher than those of GAC, except for SUC. With the aim of removing the two persistent ASs, ACE and SUC from wastewater effluent, NDMP resin proved more efficient in the removal of ACE, but GAC was more efficient at removing SUC.

### Removal of ASs in UV/AOPs

#### Laboratory-scale removal test of ASs in UV and UV/AOP

The removal of ASs by laboratory-scale UV degradation was slightly higher than the removal in WWTP-1 and WWTP-2 using post-UV disinfection units (S2 Fig). When the solution spiked with 100 μg/L of each AS was irradiated, only ACE could be removed (17% in 5 minutes). The reason may be that SUC, SAC and CYC have low molar extinction coefficients [31] and ACE can undergo direct photolysis in the UV region. The degradation of ACE is pseudo-first-order with different initial concentrations, and higher initial concentrations of ACE decrease the rate constant. The addition of other ASs also decreases the rate constant of ACE due to reduced photons scavenged by other ASs.

As the effect of regular UV disinfection to remove ASs is limited, UV/AOPs have been evaluated to degrade the micropollutants. SUC was extensively tested in various AOPs due to its strong persistence in the environment [26]. Very few studies have compared the degradation of other ASs with SUC in UV/AOPs. In this study, all four ASs were tested in two different UV/AOPs with a serial oxidant concentrations. To simulate the actual condition of raw wastewater, a pH of 7 was selected throughout the whole experiment. All four ASs followed pseudo-first-order kinetics in the UV/H_2_O_2_ process, and their reaction constants *k* (s^−1^) were in the order of ACE (1.4 × 10^−3^) > CYC (8.05 × 10^−4^) > SAC (3.59 × 10^−4^) > SUC (2.48 × 10^−4^) when 34 mg/L H_2_O_2_ was added (S3 Fig). SUC has been confirmed to have among the lowest reaction rates with OH compared to other emerging contaminants and has been used as an ideal probe for UV-based AOPs [18]. Similar to the widely acknowledged persistent contaminants ACE and SUC, the reaction rate with OH of CYC and SAC also proved to be limited (even below that of ACE).

Oxidation of ASs by UV/ H_2_O_2_ and UV/PDS exhibits similar trends. As seen in S3 Fig, degradation of ASs also followed pseudo-first-order kinetics in UV/PDS oxidation and the rate constants *k* (s^−1^) decreased in the order of ACE (5.61 × 10^−3^) > CYC (2.67 × 10^−3^) > SAC (7.23 × 10^−4^) > SUC (6.71 × 10^−4^). ACE and CYC preferred to react with OH and SO_4_^−^, while SAC and SUC had similar reaction kinetics. UV/PDS is more efficient at degrading ASs than UV/H_2_O_2_ with the same oxidant dosage. In comparison with the redox potential of OH (2.7 V), SO_4_^−^ generated from UV/PDS has a higher redox potential (2.5–3.1 at neutral pH) and higher selectivity to organic compounds [19]. Kwon et al. [32] observed a higher removal of emerging contaminants such as Bisphenol A using UV/PDS over UV/H_2_O_2_. Zhang et al. [33] found a higher removal of sulfonamide antibiotics when comparing the same processes. Xiao et al. [34] had similar results when testing disinfection byproducts, with UV/PDS giving a superior performance to UV/H_2_O_2_.

**Fig 6:**
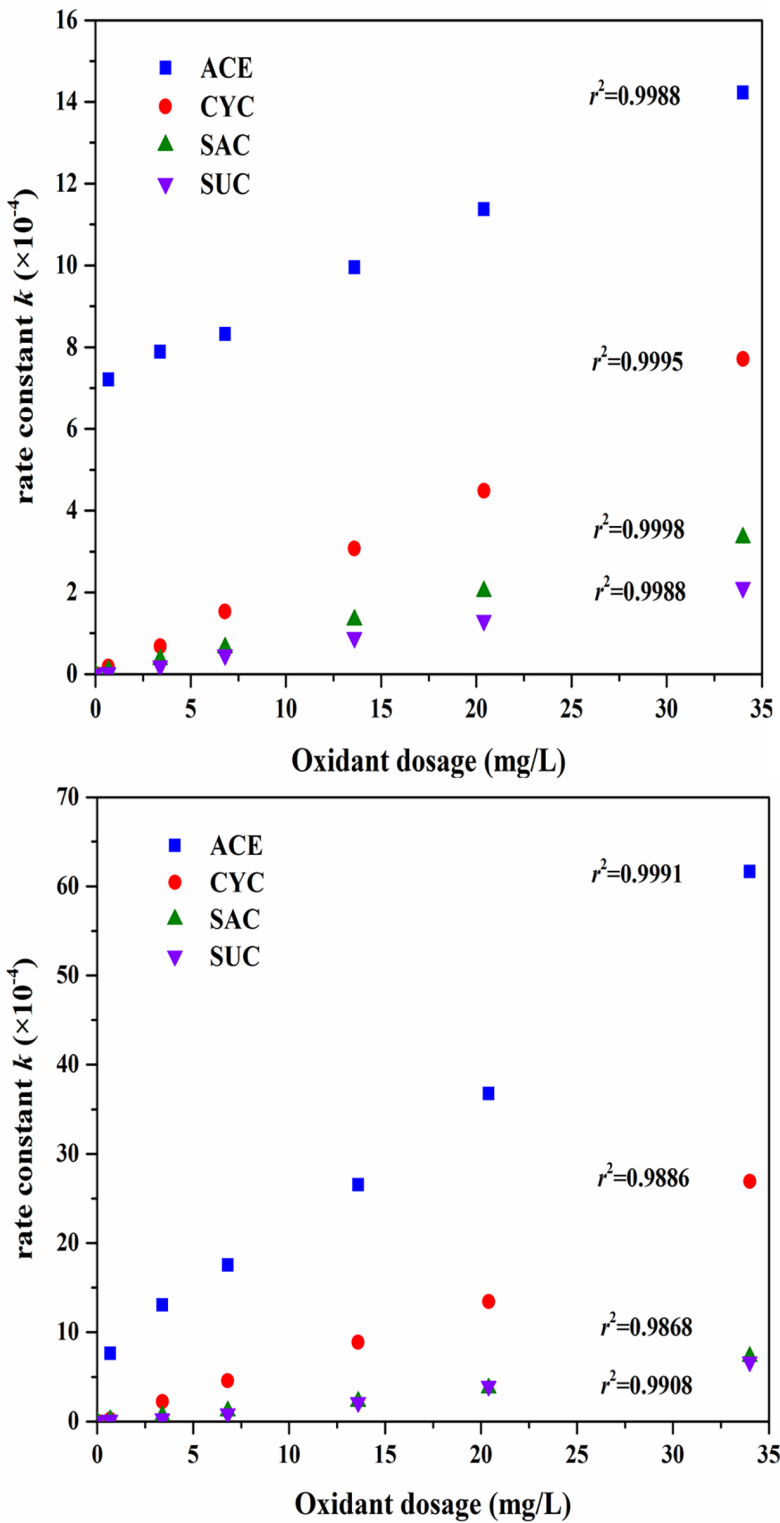
**Effect of oxidant dosage on the degradation of ASs in (a) UV/H_2_O_2_ and (b) UV/PDS (ASs: 100 μg/L, pH = 7, 30 min UV irradiation)**.

To determine the most economic oxidant dosage in both UV/AOP processes under pH = 7, a series of oxidant dosages (0.68~34 mg/L, which was far beyond the ASs in molar concentration) was tested under 30 min of UV irradiation. As shown in Fig 6, the rate constant *k* of all four ASs increased significantly with the addition of oxidants due to quicker production of OH and SO_4_^−^. The rate constants are extremely sensitive to the initial oxidant concentration, since a linear relationship between *k* and the oxidant dosage was observed, which was also found in treating other emerging contaminants such as antipyrine [35]. Usually there is a most efficient oxidant concentration with UV/AOP because excess oxidants scavenge the generated radicals as follows [36]:

$$H_{2}O_{2}+∙OH\to ∙HO_{2}+H_{2}O$$ (2)

$$S_{2}{O_{8}}^{2-}+∙SO_{4}{}^{-}\to ∙S_{2}O_{8}{}^{-}+S{O_{4}}^{2-}$$ (3)

However, no such negative impact of oxidants was found in both UV/AOPs with an oxidant concentration of up to 34 mg/L. This might be explained by the slow consumption of radicals, which means that the concentration was still below the threshold oxidant level. At any specific oxidant dose, UV/PDS achieves a faster degradation rate than UV/H_2_O_2_. Furthermore, these two UV/AOP oxidants were compared for treating secondary effluent with 34 mg/L oxidant dosage, and an economic comparison was evaluated.

#### Removal test using secondary effluent

Degradation of ASs by UV/H_2_O_2_ and UV/PDS was conducted using the secondary effluent from a full-scale WWTP to validate the results under more realistic conditions. At oxidant concentrations of 34 mg/L, the rate constant of all four ASs increased significantly, and there were no excess oxidants scavenging the generated radicals [36]. So the effluent was labeled as 100 μg/L ASs, and the amount of added oxidant was 34 mg/L. As seen in S8 Table, the rate constant *k* of ASs was 2.1–40.72 × 10^−4^ s^−1^ in UV/PDS and 0.9–6.7 × 10^−4^ s^−1^ in UV/H_2_O_2_. The rate constant *k* was reduced to a similar extent between 52.55 and 68.7% for CYC, SAC and SUC compared to that in pure water solution. As for ACE, its constant *k* was reduced by 52.14% in UV/H_2_O_2_ but by only 27.42% in UV/PDS. This difference was due to numerous inorganic ions, radical scavengers and photoresistive materials in the secondary effluent. The order of rate constants of ASs in wastewater is the same as that in laboratory-scale tests. Generally, a similar reduction in AS removal in secondary effluent (compared with that in pure water solution) was found in both UV/AOPs, and the higher rate constant *k* in UV/PDS made it more applicable to real WWTP conditions. Two reasons may account for this difference. First, SO_4_^−^ may convert to more selective halogen and carbonate radicals in wastewater, contributing to a better efficiency for UV/PDS [36]. Second, the water matrix components had a higher scavenging effect in OH oxidation than in SO_4_^−^ oxidation. This higher scavenging effect in UV/H_2_O_2_ was also reported when comparing these two UV/AOPs for degrading Bisphenol A, sulfamethoxazole, carbamazepine and other emerging contaminants [37]. Anions (mostly Cl^−^) might contribute most to the scavenging [36].

In the economic comparison, the total cost was composed of electrical energy cost and oxidant cost for the conditions of 30 min of UV irradiation and 1 mM oxidant dosage. The electrical cost was calculated to be 17.6 kWh/m^3^. The costs of H_2_O_2_ and PDS were $0.262/mol and $1.035/mol, respectively. The total cost ($/m^3^) per order was calculated by the total cost of one order of magnitude of ASs removal. It was found that the total cost of UV/PDS was much lower than that of UV/ H_2_O_2_ to achieve the same ASs removal (S8 Table). Thus, UV/PDS was recommended as the best process.

## Conclusions

In summary, ACE was only partly biodegraded after biodegradation, and A^2^/O achieved the highest removal. The anoxic and aerobic processes removed the main part of ACE, and a prepositive anaerobic process decreased the removal of ACE. Disinfection by ClO_2_ or UV produced limited removal of ASs, especially ACE and SUC. Both the adsorption and UV/AOP process could control the concentration of ASs in the environment. Resin performed better than GAC at removing ASs, with 3.33–18.51 times higher adsorption (except for SUC), while GAC was preferable in the removal of SUC. UV/PDS showed a much higher AS degradation ability and lower total cost than UV/H_2_O_2_, making it a promising method to control AS discharge into the aquatic environment.

## Supporting information

S1 Table Detailed process parameters of the investigated WWTPs. (XLSX)

S2 Table Physico-chemical properties of wastewater from sample sites. (XLSX)

S3 Table Parameters for MRM acquisition of target ASs. (XLSX)

S4 Table Instrumental and method validation data. (XLSX)

S5 Table Mean concentration of ASs along treatment process in January, April and July in five WWTPs. (XLSX)

S6 Table Estimated adsorption of ASs by GAC and resin in five full-scale WWTPs. (XLSX)

S7 Table Curve-fitting equation for removal of ASs by GAC and Resin. (XLSX)

S8 Table Efficiency and cost comparison of UV/AOP for treating ASs in secondary effluent. (XLSX)

S1 Fig Adsorption of ASs by GAC (a: C_0_ = 500 μg/L, b: C_0_ = 1000 μg/L) and resin (c: C_0_ = 500 μg/L, d: C_0_ = 1000 μg/L). (TIF)

S2 Fig Degradation kinetics of ASs in lab-scale UV disinfection. (TIF)

S3 Fig Degradation kinetics of ASs in a: UV/H_2_O_2_ and b: UV/PDS (ASs: 100 μg/L, oxidant: 34 mg/L, pH = 7). (TIF)
