# Correction: Fate of artificial sweeteners through wastewater treatment plants and water treatment processes

**DOI:** 10.1371/journal.pone.0198958

**Published:** 2018-06-07

**Authors:** Shaoli Li, Yuhang Ren, Yingying Fu, Xingsheng Gao, Cong Jiang, Gang Wu, Hongqiang Ren, Jinju Geng

In the Funding section, the grant number from the funder Jiangsu Natural Science Foundation is listed incorrectly. The correct grant number is: No. BK20161474.
