# Supplementary figures and images for: Fate of artificial sweeteners through wastewater treatment plants and water treatment processes
Source: PLoS One. 2018 Jan 2;13(1):e0189867. doi: 10.1371/journal.pone.0189867 (PMC5749728; doi:10.1371/journal.pone.0189867)

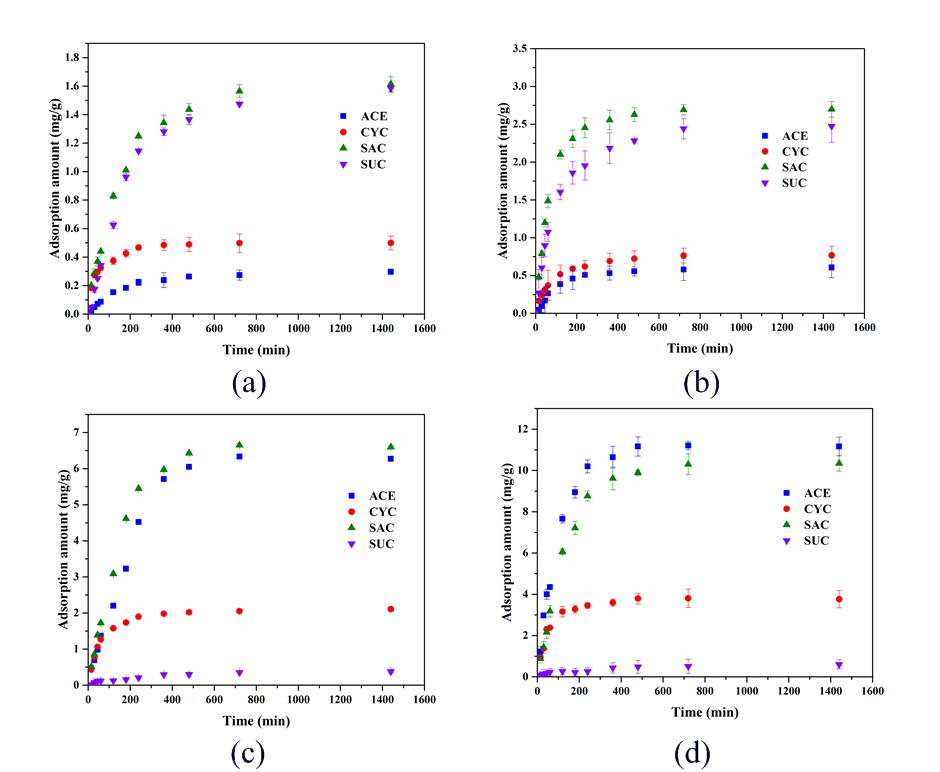

Supplement: S1 Fig — (TIF) [file pone.0189867.s009.tif]

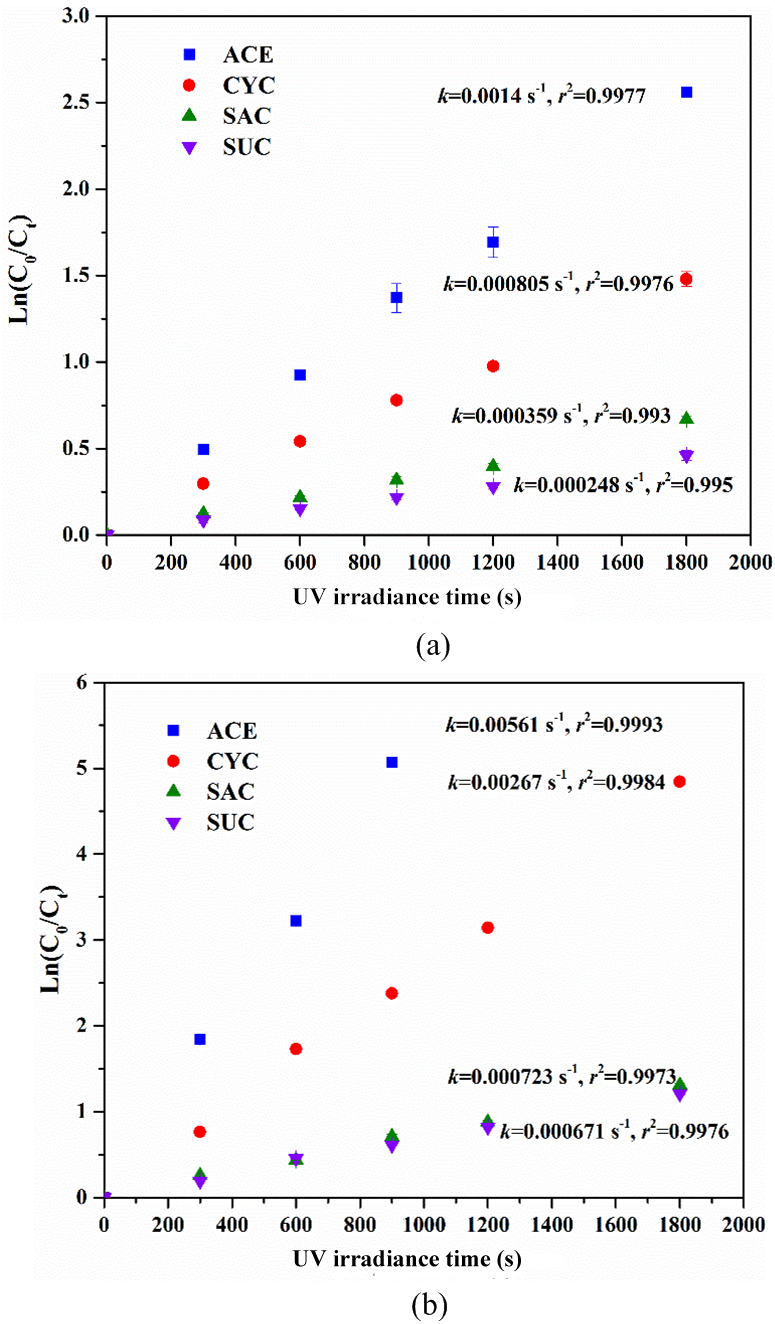

Supplement: S3 Fig — (TIF) [file pone.0189867.s011.tif]
